# Real-World Treatment Patterns and Clinical Outcomes in Patients With Stage III Non-Small-Cell Lung Cancer: Results of KINDLE-Vietnam Cohort

**DOI:** 10.3389/fonc.2022.842296

**Published:** 2022-05-23

**Authors:** Tu Van Dao, Tuan Bao Diep, Tri Le Phuong, Reto Huggenberger, Amit Kumar

**Affiliations:** ^1^Cancer Research and Clinical Trials Center, Vietnam National Cancer Hospital, Hanoi, Vietnam; ^2^Oncology Department, Hanoi Medical University, Hanoi, Vietnam; ^3^Ho Chi Minh City Oncology Hospital, Ho Chi Minh City, Vietnam; ^4^Medical Affairs, AstraZeneca Vietnam, Ho Chi Minh, Vietnam; ^5^Medical Affairs, AstraZeneca, Baar, Switzerland; ^6^Medical Affairs, AstraZeneca India, Bangalore, India

**Keywords:** lung cancer, stage III NSCLC, sequential chemoradiation, chemotherapy, chemoradiotherapy

## Abstract

**Objective:**

KINDLE-Vietnam was a part of a real-world KINDLE study with an aim to characterise treatment patterns and clinical outcomes of patients with stage III non-small cell lung cancer (NSCLC).

**Materials and Methods:**

Retrospective data from patients diagnosed with stage III NSCLC (American Joint Committee on Cancer, 7^th^ edition) between January 2013 and December 2017 with at least 9 months of follow-up were collected from 2 centres in Vietnam. Descriptive statistics were used to summarise demographics, disease characteristics and treatment modalities. Kaplan-Meier methodology evaluated survival estimates; 2-sided 95% confidence intervals (CIs) were computed. Inferential statistics were used to correlate clinical and treatment variables with median progression-free survival (mPFS) and median overall survival (mOS).

**Results:**

A total of 150 patients (median age: 60 years [range 26-82]) were enrolled; 75.3% were male, 62.0% had smoking history, 56.4% had stage IIIB disease and 62.5% had adenocarcinoma. The majority of the cases (97.3%) were not discussed at a multidisciplinary team meeting. Overall, chemotherapy alone (43.3%), radiotherapy alone (17.0%), sequential chemoradiation (13.5%) and concurrent chemoradiation (12.8%) were preferred as initial therapy. Surgery-based treatment was administered in limited patients (stage IIIA, 10%; stage IIIB, 1.3%). Palliative therapy was the most commonly administered treatment upon relapse in the second-and third-line setting. The mPFS and mOS for the Vietnam cohort were 8.7 months (95% CI, 7.59-9.72) and 25.7 months (95% CI, 19.98-42.61), respectively. The mPFS and mOS for stage IIIA were 11.9 months (95% CI, 8.64-14.95) and 28.2 months (95% CI, 24.15-not-calculable) and for stage IIIB were 7.8 months (95% CI, 6.64-8.71) and 20.0 months (95% CI, 13.01-42.61).

**Conclusions:**

KINDLE-Vietnam offers insights into the clinical findings of stage III NSCLC. There is a high unmet need for identifying patients in the early stages of NSCLC. Strategies for improving clinical outcomes in this patient population include physician education, multidisciplinary management and catering to increased access to novel agents like immunotherapy and targeted therapy.

## Introduction

Lung cancer is the most common malignancy and the leading cause of cancer-related deaths worldwide (18.4% of total cancer deaths, GLOBOCAN 2018) (1). The burden of lung cancer is extremely diverse in Asian countries. The highest incidence was observed in Japan followed by countries closer to Europe such as Armenia, Turkey and Kazakhstan as well as South-East Asia and Korea, whereas the lowest incidence was reported in Western Asian countries including Yemen and Saudi Arabia (2). South-East Asia reported 160,068 new cases and 146,990 deaths in 2018 with 23,667 new cases and 20,710 deaths reported from Vietnam (3). Although the incidence of lung cancer in Vietnam is higher than the global incidence (14.4% versus 11.6%), the mortality rate is similar to the rest of the world (18.4%) (4, 5). In the Association of South-East Asian Nations countries, the age-standardised mortality rates (per 100,000) for males and females were highest in Armenia (58.5 and 8.5), Vietnam (35.4 and 11.1) and Singapore (41.5 and 17.2) and lowest in Cambodia (21.6 and 8.7) and Indonesia (19.4 and 6) (6). The age-adjusted death rate was estimated at 24.73 per 100,000 for both genders together, putting Vietnam at the 37^th^ position in the world for lung cancer (7).

Non-small-cell lung cancer (NSCLC) accounts for about 85% of all new lung cancer cases (8). Approximately 25% to 30% of patients with NSCLC are diagnosed at stage III, described as a heterogeneous disease with either locally advanced tumour spread and/or mediastinal lymph node involvement, without clinical evidence of distant spread (9–12). Long-term survival is generally poor in stage III disease, with a 5-year median overall survival (mOS) of 36%, 26% and 13% for stage IIIA, IIIB and IIIC, respectively (13). In Vietnam, more than 80% of cases of lung cancer were NSCLC and the majority of cases (about 89%) were found in the advanced stages (IIIB or IV) (14). A study from 2014 estimated the economic burden of NSCLC in Vietnam to be >3,517 billion Vietnamese Dong, equivalent to $150 million (15). In Asia, epidermal growth factor receptor (EGFR) mutation rates in patients with NSCLC are high with approximately 47.0% in Eastern and South-Eastern Asia. Vietnam has the highest rate of EGFR mutations at 64.0% (8).

Due to heterogeneity, optimum management of stage III NSCLC remains a challenge. Surgical resection, though the preferred treatment, might not be plausible in all patients with stage III disease (16). Hence, a multi-modal management approach involving surgery, radiation and systemic agents is frequently practised. Concurrent platinum-based chemotherapy (CT) and radiotherapy (RT) are the standard of care for unresectable stage III disease with mOS ranging from 15 to 29 months (12, 17–24). Recent studies have combined immune therapy with concurrent CT and RT (cCRT), resulting in the emergence of new multi-modal combination approaches for stage III NSCLC (25, 26). Durvalumab consolidation therapy for patients with unresectable/inoperable stage III NSCLC who have not progressed on ≥2 cycles of cCRT is the new standard of care (12).

Given the significant burden of NSCLC leading to increased economic impact in Vietnam, cost-effective strategies and wide coverage are needed to manage NSCLC cases (15). A systematic and evidence-based approach to cancer care, particularly lung cancer, is a priority for Vietnam’s healthcare system (5). The data about prevalent treatment patterns and their associated survival outcomes for stage III NSCLC in the Vietnamese population is limited (27).

Vietnam was a part of the real-world evidence study KINDLE, conducted at 100 centres from 19 countries across Asia, Africa, the Middle East and Latin America (28). The primary objective of the global KINDLE study was to determine the treatment patterns and clinical outcomes of patients with stage III NSCLC, as specified by the American Joint Committee on Cancer (AJCC) criteria (7^th^ edition) in the pre-immuno-oncology era. Here we present data for stage III NSCLC in the Vietnamese population to determine the treatment patterns and clinical outcomes in patients.

## Materials and Methods

### Study Design

Retrospective data were collected over approximately 6 years (2013 to 2018) from the 2 largest cancer hospitals in Vietnam (one each in North and South Vietnam) on patients diagnosed with stage III NSCLC. The study protocol (NCT03725475) was approved by the independent ethics committees/institutional review boards of both the participating centres and the Ethical Review Committee, Vietnam Ministry of Health. The research was carried out in compliance with the Helsinki Declaration, the International Harmonisation Council (ICH), the Good Clinical Practices (GCP), the Good Pharmacoepidemiology Practices (GPP) and the relevant non-interventional and/or observational studies legislation. Adult patients (aged 18 years or older) diagnosed with *de novo* locally advanced stage III NSCLC (as per AJCC 7^th^ edition) between January 2013 and December 2017, with medical records available for a minimum of 9 months from the index date (date of diagnosis of stage III NSCLC), and signed written informed consent from the patient or next of kin/legal representative (in case of a deceased patient) were included in the study. Patients with an initial diagnosis of stage I to II NSCLC, who progressed to stage III, and those with concomitant cancer at the time of or within 5 years of stage III NSCLC diagnosis (except for non-metastatic non-melanoma skin cancers or *in situ* or benign neoplasms) were excluded.

Medical charts of eligible patients were reviewed and protocol-specified retrospective data were transcribed to the electronic case report forms. Data were collected from the index date until the end of the follow-up period, defined as death, the last medical record available or the end of the data collection. The following data were collected: demographics (age, gender, body mass index and smoking status), clinical characteristics (Eastern Cooperative Oncology Group [ECOG] performance status, NSCLC histology, stage as per 7^th^ edition AJCC, EGFR status and programmed cell death-ligand 1 status) and treatment patterns (modality and line of treatment). The occurrence and date of disease progression were determined from documents within the patient medical records, such as pathology reports, imaging reports, clinical notes and comments on disease progression. Progression-free survival (PFS) was defined as the time from the start of the treatment to documented disease progression or death due to any cause, whichever occurred first. The first progression interval was defined as the period between the index date and the first disease progression, and subsequent progression intervals were defined as the period between sequential progressions. For patients who received treatment, sequential treatment regimens were documented within each progression interval. Overall survival was calculated as the time from the stage III NSCLC diagnosis or start of the treatment to death from any cause.

### Statistical Analyses

Descriptive statistics were used to summarise patient demographics, disease characteristics and treatment modalities. Median survival estimates (mOS) and median PFS (mPFS) including rates of the affected patients were evaluated descriptively using the Kaplan-Meier survival curves and median survival estimates are reported along with the 2-sided 95% confidence interval (CI). Inferential statistics as correlation analyses were used to determine the correlation between various clinical and treatment variables and survival outcomes (mPFS and mOS). A p-value of less than 0.05 was considered statistically significant.

## Results

### Demographic and Clinical Characteristics

A total of 150 Vietnamese patients were included, of whom slightly more than half (52.0%) were alive at the time of data collection. The mean duration (± standard deviation [SD]) of follow-up was 17.52 (± 13.81) months. The median age (range) of patients was 60.0 (26.0-82.0) years; most (75.3%) were men and 62.0% (75/121) had a history of smoking or were current smokers. In total, 84 (56.4%) patients were diagnosed with stage IIIB disease (7^th^ edition AJCC classification). Adenocarcinoma was the most common histological type (62.5%, 90/144), followed by epidermoid or squamous cell carcinoma (26.4%, 38/144). Most (58.9%, 83/141) of the cases were initially presented to primary care physicians, while 41.1% (58/141) of patients consulted specialist care. The ECOG performance status was not available for the majority of the patients (n = 106); it was ≤1 in 75.0% (33/44) of the remaining patients. Majority of the cases (97.3%) were not discussed at a multidisciplinary team (MDT) meeting. **Table 1** describes the sociodemographic and clinical characteristics of the Vietnam cohort.

**Table 1:** Baseline sociodemographic and clinical characteristics of patients with stage III NSCLC in Vietnam.

Parameters	Number of Patients (N = 150)
Age (years), median (range)	60.0 (26.0-82.0)
Gender, Male, n (%)	113 (75.3)
BMI (kg/m^2^), median (range)	20.5 (16.0-30.0)
Tobacco Smoking^a^, n = 121, n (%)	
Current smoker	75 (61.9)
Ex-smoker	12 (9.9)
Never smoker	34 (28.1)
AJCC stage (7^th^ edition), n = 149, n (%)	
Stage IIIA	65 (43.6)
Stage IIIB	84 (56.4)
Histology type, n = 144, n (%)	
Adenocarcinoma	90 (62.5)
Epidermoid or squamous cell carcinoma	38 (26.4)
Large cell carcinoma	9 (6.2)
Other	4 (2.8)
Mixed	3 (2.1)
ECOG performance status, n = 44, n (%)	
≤1	33 (75.0)
≥2	11 (25.0)
T Stage, n = 149, n (%)	
T1a	6 (4.0)
T1b	8 (5.3)
T2a	23 (15.4)
T2b	14 (9.3)
T3	51 (34.2)
T4	45 (30.2)
TX	2 (1.3)
N Stage, n = 150, n (%)	
N0	7 (4.7)
N1	11 (7.3)
N2	70 (46.7)
N3	61 (40.7)
NX	1 (0.7)
EGFR testing, n = 25, n (%)	
EGFR no mutation	19 (76.0)
EGFR-mutated	5 (20.0)
Uncertain	1 (4.0)
To whom did the patient first present, n = 141, n (%)	
Primary care physician	83 (58.9)
Clinical oncologist	17 (12.0)
Others	41 (29.1)
Vital status, n = 150, n (%)	
Alive	78 (52.0)
Dead	72 (48.0)

Percentage was calculated based on the total number of patients available within each level; Unknown and missing data are not included; PD-L1 biomarker testing was performed in 1 patient who had negative status.

a Current smoker is defined as an active smoker; an ex-smoker is defined as having smoked regularly but stopped ≥365 days ago; Never smoker is defined as never smoked regularly.

AJCC, American Joint Committee on Cancer; BMI, body mass index; ECOG, Eastern Cooperative Oncology Group; EGFR, epidermal growth factor receptor; NSCLC, non-small cell lung cancer; PD-L1, programmed cell death-ligand 1.

### Treatment Patterns

**Figure 1:**
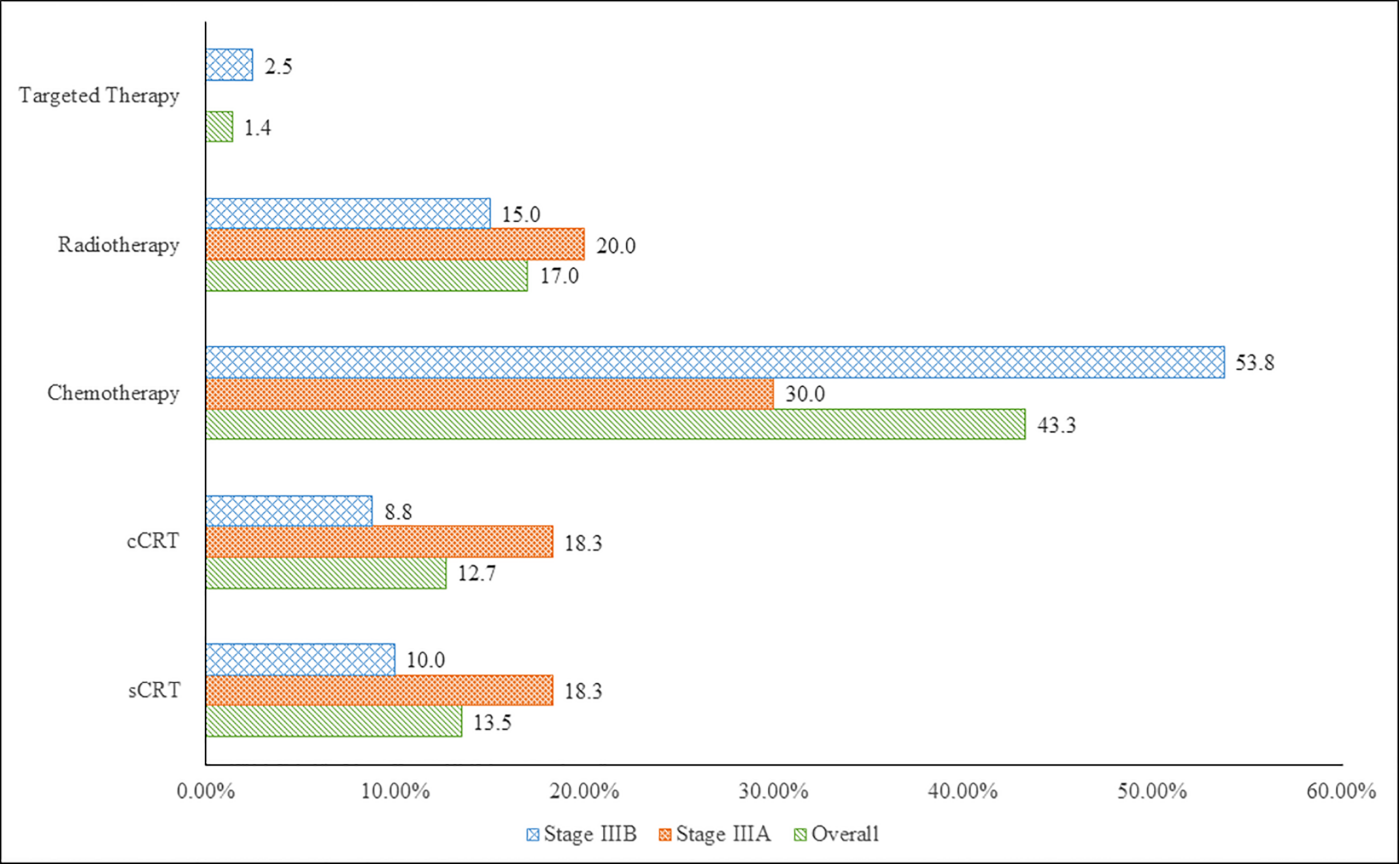
Top 5 Treatment Modalities for Stage IIIA and IIIB NSCLC in Vietnam (7^th^ Edition AJCC). cCRT, concurrent chemoradiation; sCRT, sequential chemoradiation.

Initial therapy comprised of 14 treatment modalities, including CT alone (43.3%, 61/141), RT alone (17.0%, 24/141), sequential chemoradiation (sCRT) (13.5%, 19/141), concurrent chemoradiation (cCRT) (12.8%, 18/141) and targeted therapy (1.4%, 2/141) alone or in combination with other therapies. Among the common treatment modalities reported in initial therapy in stage IIIA and IIIB disease, CT alone was administered in the majority of patients (30.0%, 18/60 and 53.8%, 43/80); RT alone (20.0%, 12/60 and 15.0%, 12/80), sCRT (18.3%, 11/60 and 10.0%, 8/80) and cCRT (18.3%, 11/60 and 8.8% 7/80) were the next common modalities (**Figure 1**). For further analyses, these treatment modalities are broadly grouped into 3 categories as surgery-based therapy, CRT-based therapy and palliative therapy (CT alone, RT alone and targeted therapy). The treatment patterns are summarised in **Table 2**. For stage IIIA and IIIB disease, palliative therapy was administered in the majority of patients (50.0% [30/60] and 71.3% [57/80]), followed by CRT-based therapy (40.0% [24/60] and 27.5% [22/80]) and surgery-based therapy (10.0% [6/60] and 1.3% [1/80]) as initial therapy. After initial therapy was administered in 141 patients (stage IIIA: 60 and stage IIIB: 80), relapse was documented for 44 (73.0%) and 73 (91.0%) patients in stage IIIA and IIIB, respectively. Second-line therapy was administered in 61 patients (stage IIIA: 29 and stage IIIB: 32), while third-line therapy was administered in 26 patients (stage IIIA: 10 and stage IIIB: 16). Palliative therapy was the most commonly administered treatment option upon relapse in second and third-line therapy (IIIA [96.6% and 100%] and IIIB [96.9% and 87.5%]).

**Table 2:** Treatment patterns in stage III NSCLC in Vietnam as per subgroup (7^th^ Edition AJCC).

Treatment Modality	Initial Treatment, n (%)			Second Line, n (%)			Third Line, n (%)		
	Overall	Stage IIIA	Stage IIIB	Overall	Stage IIIA	Stage IIIB	Overall	Stage IIIA	Stage IIIB
	n (%)	n (%)	n (%)	n (%)	n (%)	n (%)	n (%)	n (%)	n (%)
	(N = 141)	(N = 60)	(N = 80)	(N = 61)	(N = 29)	(N = 32)	(N = 26)	(N = 10)	(N = 16)
Surgery-based therapy	8 (5.7)	6 (10.0)	1 (1.3)	1 (1.6)	1 (3.4)	0	0	0	0
CRT-based therapy	46 (32.6)	24 (40.0)	22 (27.5)	1 (1.6)	0	1 (3.1)	2 (7.7)	0	2 (12.5)
Palliative therapy^a^	87 (61.7)	30 (50.0)	57 (71.3)	59 (96.7)	28 (96.6)	31 (96.9)	24 (92.3)	10 (100)	14 (87.5)
Top 5 Treatment Modalities									
sCRT	19 (13.5)	11 (18.3)	8 (10.0)	1 (1.6)	0	0	1 (3.8)	0	1 (6.3)
cCRT	18 (12.7)	11 (18.3)	7 (8.8)	0	0	0	0	0	0
CT	61 (43.3)	18 (30.0)	43 (53.8)	39 (63.9)	16 (55.2)	23 (71.9)	17 (65.4)	6 (60.0)	11 (68.8)
RT	24 (17.0)	12 (20.0)	12 (15.0)	16 (26.2)	11 (37.9)	5 (15.6)	6 (23.1)	4 (40.0)	2 (12.5)
Targeted therapy	2 (1.4)	0	2 (2.5)	4 (6.6)	1 (3.4)	3 (9.4)	1 (3.8)	0	1 (6.3)

a Includes CT alone, RT alone and targeted therapy.

AJCC, American Joint Committee on Cancer; cCRT, concurrent chemoradiation; CRT, chemoradiation; CT, chemotherapy; RT, radiotherapy; sCRT, sequential chemoradiation.

### Survival Outcomes

**Figure 2:**
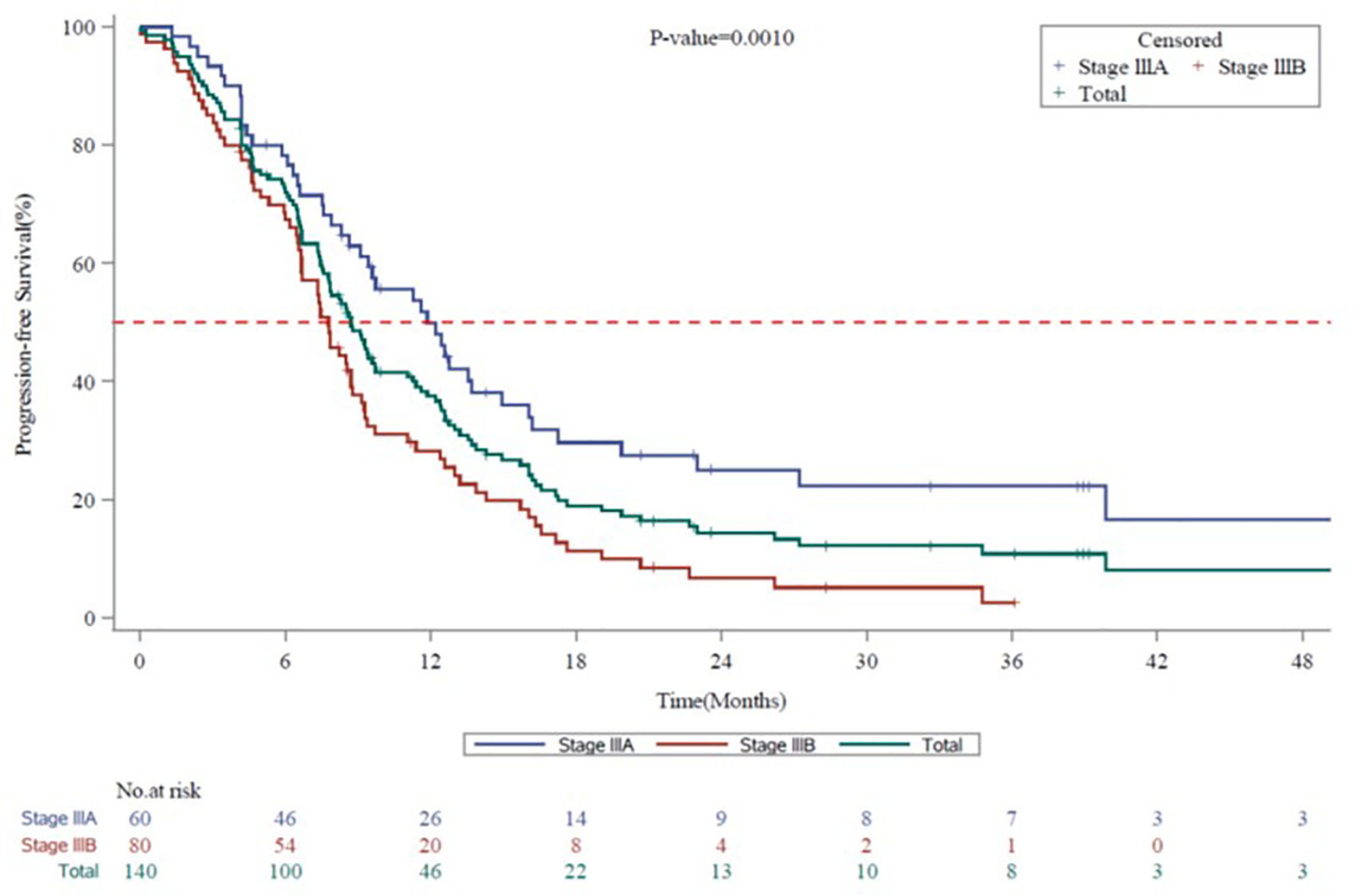
Kaplan-Meier Survival Curves for Progression-free Survival by Disease Stage (7^th^ Edition AJCC) in Vietnam. AJCC, American Joint Committee on Cancer; CI, confidence interval; mPFS, median progression-free survival. Kaplan-Meier Survival curves for progression-free survival for all stage III NSCLC patients are shown in green, whereas stage IIIA and stage IIIB patients are shown in blue or red, respectively. mPFS for the entire Vietnam cohort, 8.7 months (95% CI, 7.59 to 9.72). mPFS for stage IIIA, 11.9 months (95% CI, 8.64 to 14.95). mPFS for stage IIIB, 7.8 months (95% CI, 6.64 to 8.71).

**Figure 3:**
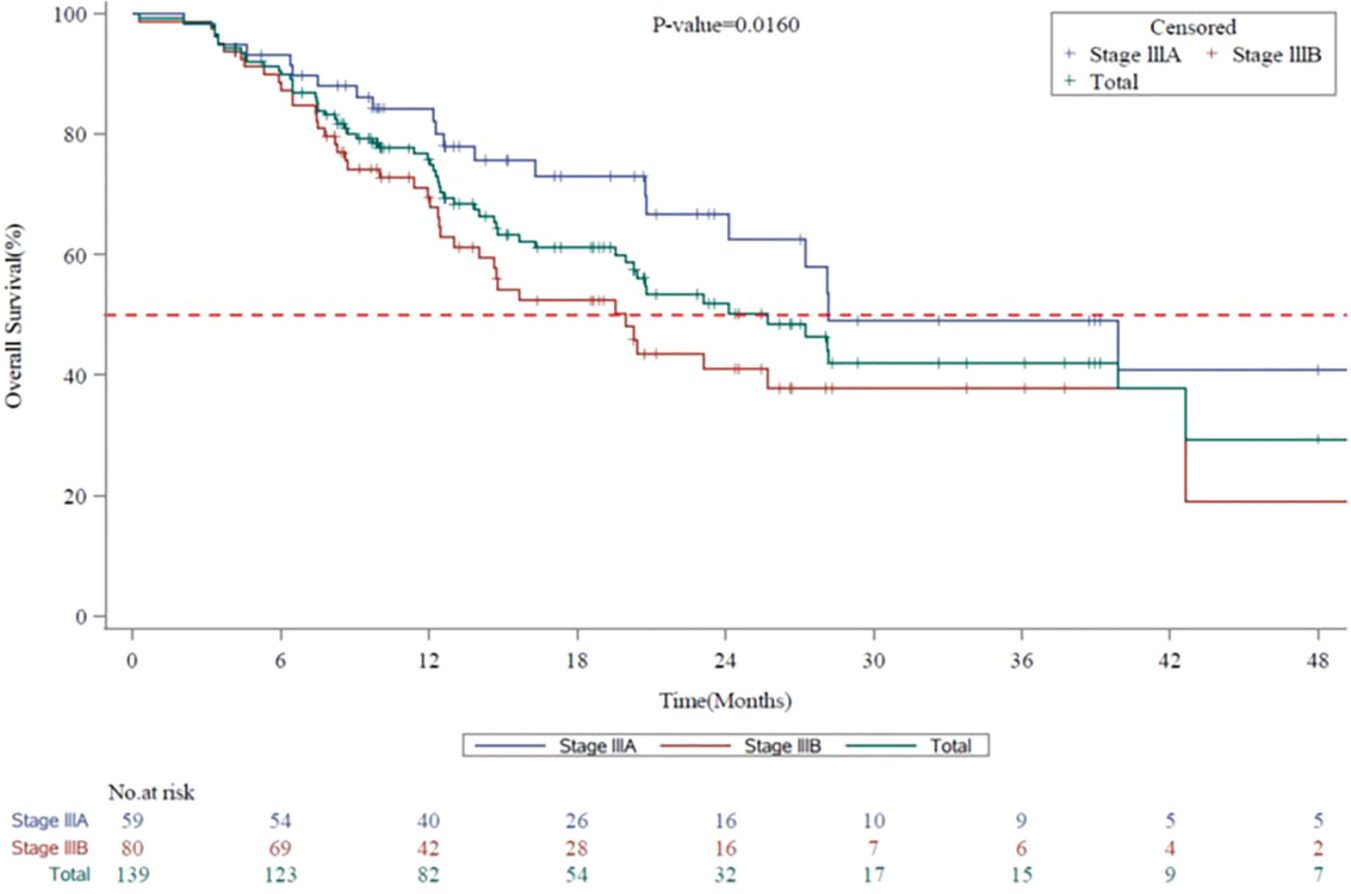
Kaplan-Meier Survival Curves for Overall Survival by Disease Stage (7^th^ Edition AJCC) in Vietnam. AJCC, American Joint Committee on Cancer; CI, confidence interval; mOS, median overall survival. Kaplan-Meier survival curves for overall survival for all stage III NSCLC patients are shown in green, whereas stage IIIA and stage IIIB patients are shown in blue or red, respectively. mOS for the entire Vietnam cohort, 25.7 months (95% CI, 19.98 to 42.61). mOS for stage IIIA, 28.2 months (95% CI, 24.15 to not-calculable [NC]). mOS for stage IIIB, 20.0 months (95% CI, 13.01 to 42.61).

The mPFS for the entire evaluable population (N=140) was 8.7 months (95% CI, 7.59 to 9.72): stage IIIA, 11.9 months (95% CI, 8.64 to 14.95) and stage IIIB, 7.8 months (95% CI, 6.64 to 8.71) (**Figure 2**). The mOS for the entire population evaluable in 139 patients was 25.7 months (95% CI, 19.98 to 42.61): stage IIIA, 28.2 months (95% CI, 24.15 to not-calculable [NC]) and stage IIIB, 20.0 months (95% CI, 13.01 to 42.61) (**Figure 3**). The mPFS was numerically higher in patients who underwent surgical resection (n = 18; 9.7 months [95% CI, 6.31 to 13.57]) compared with unresectable patients (n = 91; 8.7 months [95% CI, 6.67 to 12.19]). The mOS was NC in patients who underwent surgical resection (n = 18; NC [95% CI, 13.83 to NC]), while in unresectable patients it was calculable (n = 90; 23.1 months; [95% CI, 14.65 to 39.89]).

The survival outcomes according to the initial therapy are described in **Table 3**. In stage IIIA, the longest mPFS and mOS were observed for patients who underwent CRT-based therapy (n = 24; 13.7 months [95% CI, 8.31 to 16.20] and 28.1 months [n = 23; 95% CI, 20.76 to 39.89]), followed by patients who underwent surgery-based therapy (n = 6; 9.7 months [95% CI, 4.14 to 51.98] and 13.8 months [n = 6; 95% CI, 9.10 to NC]). In stage IIIB, the longest mPFS was observed in a single patient who underwent surgery-based therapy (20.7 months [95% CI, NC to NC]), followed by CRT-based therapy in 22 patients (8.6 months [95% CI, 6.70 to 12.39]). In stage IIIB, the longest mOS of 23.1 months (n = 22; 95% CI, 8.21 to 42.61) was observed for CRT, followed by palliative therapy (n = 57; 19.5 months [95% CI, 13.01 to 42.61]).

**Table 3:** Survival outcomes in stage III NSCLC in Vietnam as per Initial treatment regimen and stage (7^th^ Edition AJCC).

	mPFS (95% CI), Months		mOS (95% CI), Months	
	Stage IIIA	Stage IIIB	Stage IIIA	Stage IIIB
	(N = 60)	(N = 80)	(N = 59)	(N = 80)
Surgery-based therapy	9.7 (4.14 to 51.98)	20.7 (NC to NC)	13.8 (9.10 to NC)	NC (NC to NC)
CRT-based therapy	13.7 (8.31 to 16.20)	8.6 (6.70 to 12.39)	28.1 (20.76 to 39.89)	23.1 (8.21 to 42.61)
Palliative therapy^a^	9.6 (6.31 to 12.58)	7.4 (5.95 to 8.71)	NC (27.24 to NC)	19.5 (13.01 to 42.61)

a Includes CT alone, RT alone and targeted therapy.

AJCC, American Joint Committee on Cancer; CI, confidence interval; CRT, chemoradiation; CT, chemotherapy; mPFS, median progression-free survival; mOS, median overall survival; NC, not calculable; RT, radiotherapy.

Univariate analysis for mPFS and mOS favoured stage IIIA (hazard ratio [HR], 0.53, 95% CI, 0.36 to 0.78; p = 0.001) and (HR, 0.53, 95% CI, 0.31 to 0.90; p = 0.016) compared with stage IIIB. The details of univariate analysis as per mPFS and mOS for stage IIIA and IIIB disease for clinico-demographic characteristics and treatment modalities are mentioned in **Table 4**. As per the univariate analyses, for stage IIIA, female gender and surgery as a treatment modality were associated with better mPFS, while adenocarcinoma was associated with better mOS (p < 0.05). For stage IIIB disease, adenocarcinoma was associated with better mOS (p < 0.05).

**Table 4:** Univariate analyses for survival outcomes in stage III NSCLC in Vietnam based on clinico-demographic characteristics and treatment regimen (7^th^ Edition AJCC).

Characteristics	Stage IIIA		Stage IIIB	
	HR (95% CI)	p value	HR (95% CI)	p value
Univariate Analyses for PFS				
Age >65 vs ≤65	1.28 (0.73 to 2.22)	0.3833	0.59 (0.33 to 1.06)	0.0762
Male vs Female	1.82 (1.06 to 3.12)	**0.0285**	0.85 (0.48 to 1.48)	0.5675
Adenocarcinoma vs Others	0.85 (0.52 to 1.39)	0.5265	0.70 (0.42 to 1.17)	0.1720
Surgery in initial therapy yes vs no	0.23 (0.09 to 0.57)	**0.0014**	0.20 (0.03 to 1.46)	0.1114
CRT in initial therapy yes vs no	0.63 (0.24 to 1.67)	0.3526	0.43 (0.04 to 4.85)	0.4954
Palliative therapy^a^ in initial therapy yes vs no	0.67 (0.22 to 2.09)	0.4947	0.45 (0.04 to 5.28)	0.5234
Univariate Analyses for OS				
Age >65 vs ≤65	1.20 (0.59 to 2.43)	0.6161	0.95 (0.50 to 1.78)	0.8650
Male vs Female	1.57 (0.79 to 3.11)	0.1989	1.23 (0.63 to 2.40)	0.5359
Adenocarcinoma vs Others	0.53 (0.29 to 0.99)	**0.0464**	0.57 (0.32 to 0.99)	**0.0451**

a Includes CT alone, RT alone and targeted therapy.

AJCC, American Joint Committee on Cancer; CI, confidence interval; CRT, chemoradiation; CT, chemotherapy; HR, hazard ratio; OS, overall survival; PFS, progression-free survival; RT, radiotherapy.

Values in bold indicate statistically significant difference (p < 0.05).

## Discussion

To our knowledge, this is the first real-world study reporting the treatment patterns and their associated clinical outcomes in a group of Vietnamese patients with stage III NSCLC. This research offers an overview of treatment and survival trends of unresectable/inoperable stage III NSCLC. MDT discussion in lung cancer is known to be associated with better treatment decisions, which potentially improves outcomes and quality of life for patients with lung cancer (29, 30). In our study, a majority (97.3%) of the cases were not discussed at MDT meetings. Although both the participating centres are multi-speciality cancer care (including medical oncologists, radiologists, pathologists and thoracic surgeons), MDT management of lung cancer was not established as part of routine clinical practice in Vietnam during the period of this study.

A recent study assessing real-world clinical outcomes in Medicare patients reported a higher (61.0%) proportion of patients being treated with CRT in stage IIIA and 39.0% in stage IIIB (22, 31). With almost 14 treatment modalities being used as initial therapy, our study observed a wide difference in the treatment trends. More than half of the patients (61.7%) underwent palliative therapy as initial treatment. The American Society of Clinical Oncology guidelines recommend early palliative care to improve symptoms, quality of life and increase survival. Palliative care for NSCLC is categorised into 2 major groups, namely, supportive care and tumour-directed treatment (includes palliative CT and RT) (32). As initial therapy, CT alone was the dominant therapy (43.3%), showing that patients had limited access to RT, given to only 17.0% of the patients. In 2017, intensity-modulated radiation therapy was sparingly used and only 36 linear machines were available in Vietnam for external radiation therapy with 3D-conformal radiation therapy techniques in the country resulting in a low rate of RT and CRT (33). As second- or third-line therapies upon relapse, palliative therapy was the most preferred option in Vietnamese patients and rather the only option administered in the third-line therapy in stage IIIA NSCLC, whereas in KINDLE-global, CT alone was the most favoured second- or third-line therapy after recurrence, followed by RT alone in >20% of patients (28).

Our Vietnam data reports statistically significant longer mPFS (11.9 versus 7.8 months; HR 0.53 [95% CI, 0.36 to 0.78]; p = 0.001) and mOS (28.2 versus 20.0 months; HR 0.53 [95% CI, 0.31 to 0.90]; p = 0.016) in stage IIIA as compared with stage IIIB disease. Similar results were also observed in the KINDLE-global cohort, where longer mPFS (14.3 versus 10.2 months; HR 0.86 [95% CI, 0.77 to 0.96]; p < 0.01) and mOS (43.8 versus 27.7 months; HR 0.78 [95% CI, 0.68 to 0.90]; p = 0.0005) were observed in stage IIIA than stage IIIB [28]. In a Vietnamese study, 51 patients with advanced NSCLC underwent first-line combination CT with pemetrexed and cisplatin followed by pemetrexed maintenance; mPFS and mOS were reported to be 7.8 months and 16.1 months, respectively (34). In our study, CRT-based therapy prolonged mPFS (13.7 and 8.6 months) and mOS (28.1 and 23.1 months) irrespective of stage IIIA or IIIB. Similar results were seen in a retrospective Korean study where unresectable stage III patients treated with CRT had better mOS (30.3 months) (95% CI, 26.6 to 34.0) compared with palliative therapy (14.7 months) (95% CI, 13.0 to 16.4) (35).

Real-world data about the adherence to guideline-directed therapies and patient outcomes especially from low-and-middle-income countries are limited. Several randomised clinical studies have also shown a beneficial effect of cCRT compared with sCRT or RT alone in unresectable patients (20–22). In a retrospective study from Vietnam, 5,220 patients with stage III lung cancer were analysed over 3 years of which 70 stage III lung cancer patients having valid survival information were identified (11.7%). The 3-year survival probability of patients with surgery and/or CRT was 34.3% (16.2% to 72.4%), which was higher (10.6%) than CT or RT alone (4.9% to 23.2%; p = 0.055) (15). Another study showed a survival benefit with cCRT having better survival benefits than systemic therapy (14.7 versus 10.9 months) and RT (7.8 months) (36). The preliminary findings of PERTAIN study suggest that implementation of fluorodeoxyglucose-positron emission tomography/CT-guided cCRT results in a substantial improvement in mPFS and mOS for patients with stage III NSCLC in low-and-middle-income countries (37). As a multi-modal treatment, cCRT remains the standard of care for stage III NSCLC with proven benefits over single treatment approaches (38). However, in our study, the use of the cCRT regimen was quite low (12.7%) as initial therapy in comparison to CT alone (43.3%). The most probable reason for this practice was that the MDT meeting approach was employed for discussing <5% of the cases. The MDT (lung cancer tumour boards) requires close collaboration between medical oncologists, radiologists and thoracic surgeons to make an informed decision based on the resectability of the tumour on considering cCRT as an initial treatment modality. Improving the capacity of the radiation approach for stage III NSCLC patients is an important solution for Vietnam. Though the role of MDT is to identify eligible patients who can tolerate cCRT over other approaches, the survival gain of aggressive cCRT may be negated by its severe toxicities (39).

In univariate analysis, adenocarcinoma was a significant positive predictive factor for mOS in both stage IIIA and IIIB disease. The mOS was found to be better in patients with adenocarcinoma (p < 0.046) than in patients with other carcinomas. The female gender was associated with a significantly lower risk of death in both stage IIIA and IIIB. Several studies have shown the female gender as a good prognostic factor (40, 41). However, in one of the studies, no significant difference was found between genders in terms of survival (3-year survival, 29% versus 24%). This was attributed to a smaller proportion of patients being females and which may have influenced the statistical power (42). Adenocarcinoma has a higher association with smoking in females. The DNA adduct levels are higher among females with adenocarcinoma than their male counterparts after adjustment for smoking dose, and thus females are at higher risk as they are exposed to higher levels of tobacco carcinogens than males (43). Only a small number of patients were available with EGFR test results (n = 25) and the prevalence was found to be 20% (n = 5). Adenocarcinoma is the most frequently encountered histological type of NSCLC, accounting for about half of the cases of NSCLC (44, 45). Adenocarcinoma is also reported to have a better prognosis as compared with other histology subtypes of NSCLC.

With a 1-year survival rate of 42% and a 5-year survival rate of 16%, the survival rate of patients with lung cancer remains low in Vietnam (6). The poor prognosis reflects existing treatment gaps in the management of lung cancer in Vietnam. This KINDLE-Vietnam data provides a benchmark for understanding the treatment patterns, which will be important for evaluating the effectiveness of newer therapies in this population as they become part of clinical practice guidelines. This evidence will also support patient access in Vietnam as a majority (almost 80%) of cancer care (examination and treatment) is covered by health insurance (46).

The limitations of our study include the small sample size and the known challenges of a retrospective study design in real-world settings. Additionally, data collection was limited to the availability of existing health records, resulting in missing data, as many patients could have been lost to routine clinical follow-up. In the initial therapy setting, the study duration covers the era before immunotherapy approval. Thus, the data on the effectiveness of targeted and immunotherapy agents have not been captured in the study.

## Conclusion

KINDLE-Vietnam study describes the treatment patterns in stage III NSCLC and offers real-world insights into the therapy landscape in the Vietnamese population. Although the study notes adherence to the treatment protocols for CRT-based therapy as the initial therapy in most patients with unresectable disease, there is a definite gap in the optimal selection and sequencing of different treatment approaches. The findings also highlight the need for newer treatment options like immunotherapy in patients with unresectable disease post CRT. Concurrent CRT has been shown to produce better outcomes than sequential CRT. In addition, the PACIFIC trial established CRT followed by 1 year of durvalumab as the standard of care in patients with unresectable stage III NSCLC. Nevertheless, there remains to be a high unmet need for identifying patients in the early stages of NSCLC. Strategies for improving patient outcomes, including guideline adoption, physician education and multidisciplinary management, and catering to increased access to novel agents like immunotherapy and targeted therapy are needed. The data obtained from this study will also contribute to a consolidated framework to help understand the unmet clinical needs in Vietnam in line with their current focus of strengthening the National Cancer Control Programme initiative, in addition to providing baseline data to determine the potential effect of new therapies on the treatment of stage III NSCLC in this region.

## Data Presented At

KINDLE Global Data Poster Presented at ASCO: Jazieh AR, et al. Contemporary Management and Associated Outcomes of 3,151 Patients With Stage III Non-small Cell Lung Cancer (NSCLC) in a Real-world Setting: Results of KINDLE, a Multicountry Observational Study. J Clin Oncol (2020) 38:15_suppl, 9043-9043. KINDLE Global Data Manuscript Published: Jazieh AR, et al. Real-World Treatment Patterns and Clinical Outcomes in Patients With Stage III NSCLC: Results of KINDLE, a Multicountry Observational Study. J Thorac Oncol (2021) 16(10):1733-1744. DOI:10.1016/j.jtho.2021.05.003.

## Data Availability Statement

The original contributions presented in the study are included in the article/supplementary material. Further inquiries can be directed to the corresponding author.

## Ethics Statement

The studies involving human participants were reviewed and approved by the Ethic Committee in Biomedical Research - National Cancer Hospital, the Ethic Committee in Biomedical Research - HCM Oncology Hospital, and the National Ethic Committee in Biomedical research - Vietnam Ministry of Health. The patients/participants provided their written informed consent to participate in this study.

## Author Contributions

TVD, TBD: Conceptualization, Methodology, Writing, Investigation, Editing. TP: Conceptualization, Writing, Methodology, Visualization, Editing. RH, AK: Conceptualization, Methodology, Editing. All authors contributed to the article and approved the submitted version.

## Conflict of Interest

Authors RH, AK and TP are employed by the company AstraZeneca.

The authors declare that the KINDLE study and this manuscript writing were funded by AstraZeneca. The funder had the following involvement with the study: study design, analysis, interpretation of data, and the writing of this article.

## Publisher’s Note

All claims expressed in this article are solely those of the authors and do not necessarily represent those of their affiliated organizations, or those of the publisher, the editors and the reviewers. Any product that may be evaluated in this article, or claim that may be made by its manufacturer, is not guaranteed or endorsed by the publisher.
